# Case Report: Acute abdomen and large mesenteric mass as another face of multisystem inflammatory syndrome in an adolescent child: measure twice, act once!

**DOI:** 10.3389/fped.2023.1324670

**Published:** 2023-12-08

**Authors:** Mustafa Bakir, Umair Iqbal, Ayoolamide N. Gazal, Daniel Robertson

**Affiliations:** ^1^ Department of Pediatrics, University of Illinois College of Medicine at Peoria, Peoria, IL, United States; ^2^ Department of Surgery, University of Illinois College of Medicine at Peoria, Peoria, IL, United States

**Keywords:** COVID-19, MIS-C, appendicitis, mesenteric lymphadenopathy, Kikuchi-Fujimoto disease, Kawasaki disease

## Abstract

**Introduction:**

During the Covid-19 era, acute abdomen and mesenteric lymphadenopathy were encountered as frequent mimic of appendicitis. This phenomenon can further complicate the diagnosis of acute abdominal conditions, not solely due to bacterial or viral intestinal infections but also attributable to post-infectious acute inflammatory states resulting in either undertreatment of inflammatory conditions or unnecessary surgery.

**Case presentation:**

In this case study, we present the case of an 11-year-old female who initially presented with fever and right lower quadrant abdominal pain, raising concerns of appendicitis. However, upon further investigation, it was revealed that she harbored a sizable mesenteric mass. Subsequent biopsy results unveiled a significant necrotic mesenteric lymphadenitis. Notably, this patient fulfilled the criteria for Multisystem Inflammatory Syndrome in Children (MIS-C), a condition that manifested following persistent postoperative fever. Remarkably, the patient exhibited a highly favorable response to the treatment administered. This clinical scenario presents an atypical manifestation of MIS-C, as the patient displayed a substantial mesenteric mass alongside symptoms mimicking appendicitis, within the context of an acute abdomen.

**Conclusion:**

Clinicians should consider MIS-C and other post-infectious inflammatory conditions in mind when diagnosing acute abdominal cases. The presented case underscores the importance of recognizing atypical presentations of MIS-C that can mimic appendicitis, sometimes necessitating surgical resection of a large lymph node. We propose diagnostic flow chart to aid in the differentiation of acute bacterial appendicitis from MIS-C.

## Introduction

During the Covid-19 era, a substantial number of case reports has underscored mesenteric lymphadenopathy as a frequent mimic of acute abdomen and appendicitis. This phenomenon can further complicate the diagnosis of acute abdominal conditions, not solely due to bacterial or viral intestinal infections but also attributable to post-infectious acute inflammatory states like MIS-C, Multisystem Inflammatory Syndrome (1) secondary to Covid-19 vaccination (MIS-V), Kikuchi-Fujimoto Disease (KFD), even intra-abdominal malignancy. This discussion delves into the intricate differential diagnosis of our presented case, contemplating clinical, pathological, and immunological facets of each condition. A clinician-tailored algorithm could offer valuable guidance in distinguishing between infectious and inflammatory disorders that mimic appendicitis in the context of the COVID-19 pandemic.

## Case presentation

An 11-year-old previously healthy female presented to the emergency department with a 2-day history of aching and dull right lower abdominal pain, rated at 7/10 in intensity, along with reduced appetite and fever reaching up to 102°F. Physical examination revealed unremarkable findings, except for tenderness in the right lower quadrant of the abdomen, particularly pronounced at McBurney's point, without signs of guarding or rebound tenderness.

Her medical history indicated exposure to multiple family members with Covid-19 infection about 5 weeks prior to admission, although she was tested negative and remained asymptomatic. Notably, she had completed both doses of the Pfizer mRNA vaccine series 10 and 6 weeks before her current presentation. The patient's history yielded no indications of recent travel, contact with animals, consumption of raw meals, or insect bites. Notably, the patient resides in an urban area, further minimizing the likelihood of exposure to environmental factors commonly associated with fever of unknown source.

The patient underwent an evaluation for possible appendicitis, which included laboratory tests. The complete blood count returned unremarkable results except for lymphopenia (absolute lymphocyte count of 330/mcl (normal: 900–3,400/mcl). An automated urinalysis showed positive results for 21–30 white blood cells, 2+ blood, mild proteinuria, and sterile urine culture. The comprehensive metabolic panel remained within normal limits. Abdominal ultrasonography was hindered by gas, preventing visualization of the appendix.

After an initial discharge, the patient was readmitted the following day with a presumed diagnosis of appendicitis due to persisting right lower quadrant abdominal pain, fever, and focal tenderness in the same area, accompanied by rebound tenderness during the physical examination. PCR tests for Covid-19 and influenza A&B returned negative results. The clinical evaluation pointed towards acute appendicitis with focal peritonitis. Consequently, she underwent diagnostic laparoscopy, which revealed an 8 cm mass within the ascending mesocolon extending towards the mesentery's root.

**Figure 1:**
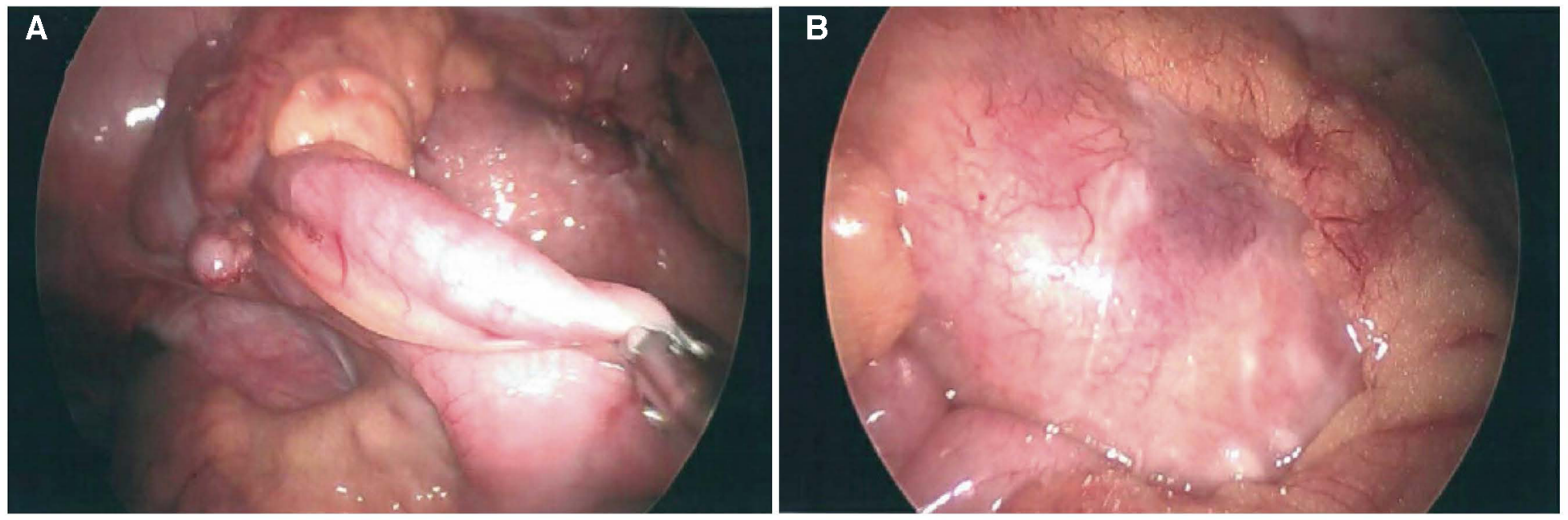
Laparoscopic images of non-perforated relatively normal appearing appendix (**A**) and an inflammatory mass in the adjacent colonic mesentery with associated fibrinopurulent exudate (**B**).

Further investigation was warranted to ascertain the nature of the mass, considering possibilities like abscess due to perforated appendicitis, inflammatory bowel disease, or neoplasm. A laparoscopic right hemicolectomy was performed, involving high ligation of the ileocolic pedicle and a stapled anastomosis, with no evidence of intestinal perforation (Figure 1). IV Ceftriaxone was administered for 2 days post-surgery until sterile peritoneal culture results. Flow cytometric analysis of the colonic mass did not indicate abnormal lymphoid cell populations.

**Figure 2:**
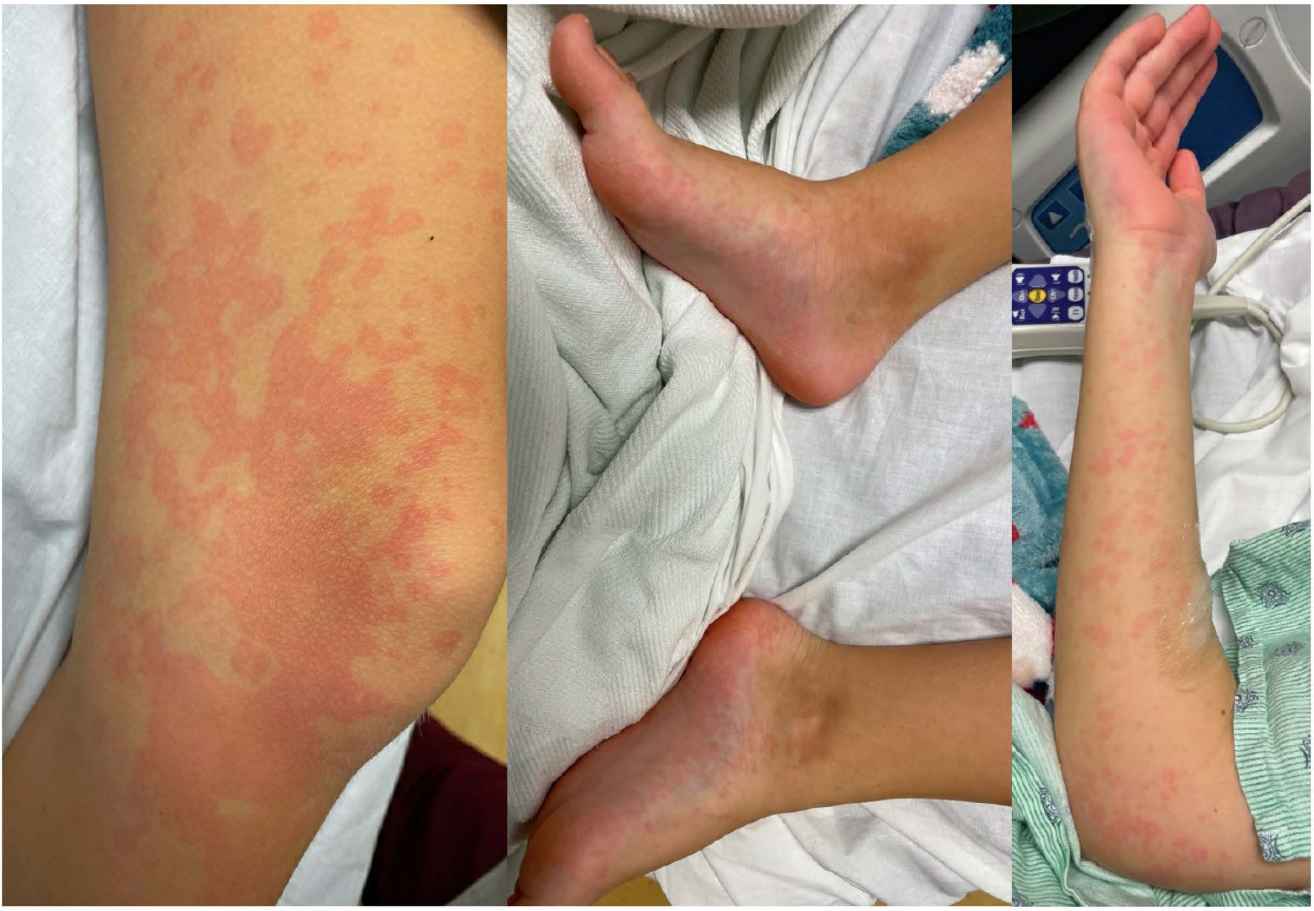
Erythematous patchy maculopapular rash involving trunk and all extremities including palms and soles.

The patient's postoperative course was marked by persistent fever, an extensive polymorphous maculopapular rash (Figure 2), bulbar conjunctival injection, and diarrhea. The respiratory pathogen PCR panel detected adenovirus and RSV. While viral or post-surgery explanations were plausible, on the fifth postoperative day, the differential diagnosis was broadened to include MIS-C, incomplete Kawasaki disease, and other infections.

Additional laboratory findings included hypoalbuminemia (2.6 g/dl, normal: 3.8–5.6 g/dl), hyponatremia (130/mmol/L (normal: 136 – 145 mmol/L), anemia (Hb 8.5 g/dl), lymphopenia (absolute lymphocyte count of 180/mm^3^), thrombocytopenia (146,000/mcl, normal: 199–367/mcl), elevated CRP (22 mg/dl, normal: <0.50 mg/dl), ESR (37 mm/h, normal: 3–13 mm/h), procalcitonin (1.33 ng/ml, normal: <0.25 ng/ml), ferritin (581 mcg/L, normal: 5–204 ng/ml), D-dimer (2.59 mcg/ml, normal: 0.50 mcg/ml), troponin (19 ng/L, normal: <17 ng/L), and positive SARS-CoV-2 IgG (antibodies against the nucleocapsid protein of SARS-CoV-2). Notable negative results included C. difficile toxin, gastric pathogen array, blood cultures, EBV (Epstein-Barr virus) and CMV (Cytomegalovirus) serology panels, and an echocardiogram.

**Figure 3:**
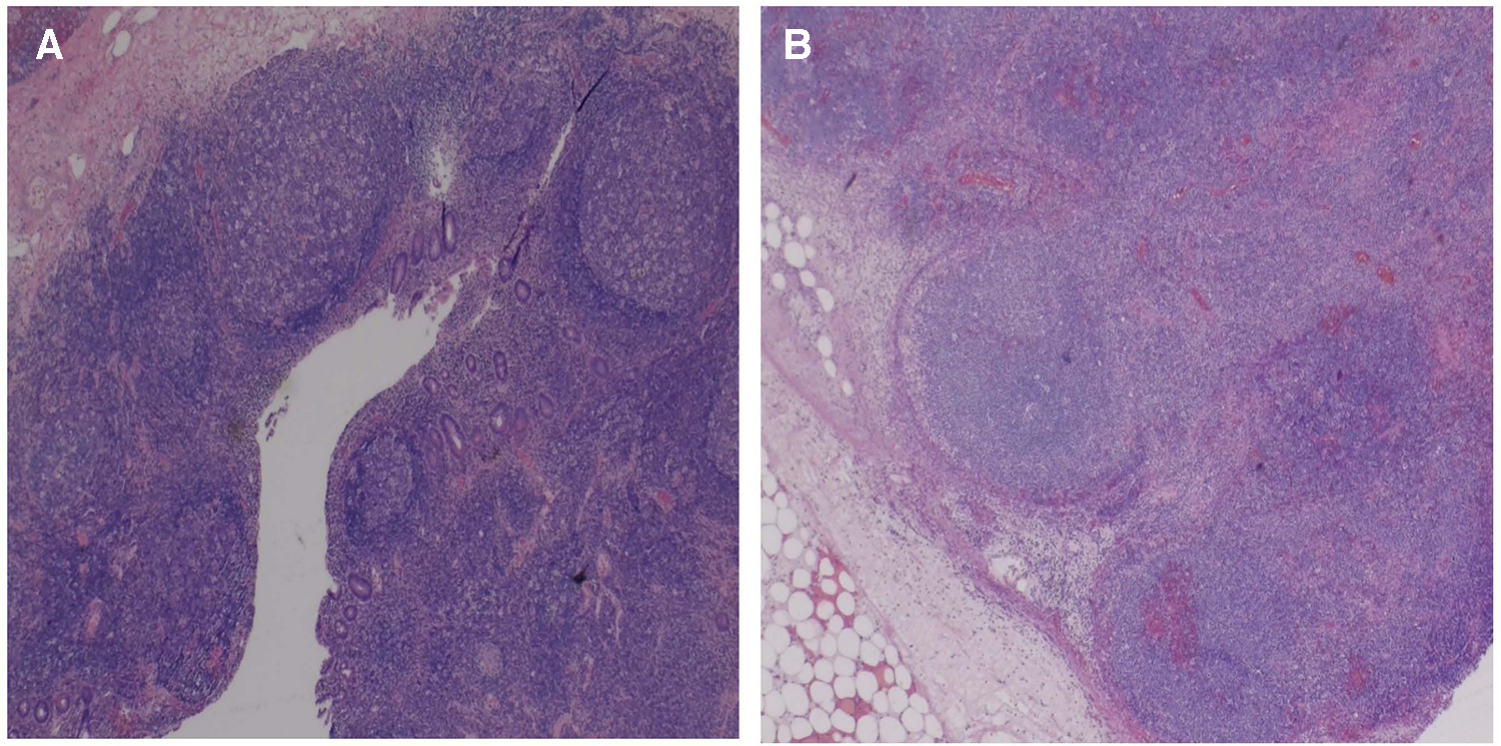
Sections of the mesenteric/pericolic mass show markedly enlarged lymph nodes with adjacent fat stranding acute inflammation, and edema (**A**), and overall maintained lymph node architecture with dilated sinuses, abundant hemorrhage, and geographic areas of necrosis (**B**).

Meeting the MIS-C criteria, treatment was initiated with IVIG at a dose of 2 gm/kg and enoxaparin. The administration of steroids was withheld pending pathology results of the colonic mass, and aspirin was postponed due to the patient's post-surgical colonic recovery. Subsequently, the patient's rash, inflammatory markers, and overall clinical condition showed significant improvement. She was discharged following 48 h of being afebrile. She remained well at 1 month of follow-up. The histopathological examination of the lymph node confirmed necrotizing non-histiocytic lymphadenitis (Figure 3).

## Discussion

We present the case of an adolescent girl whose initial symptoms suspicious for acute appendicitis, later progressed with persistent fever, a distinctive skin rash, and conjunctival injection after her laparoscopic removal of a mesenteric mass with hemicolectomy that entailed broadening of differential diagnosis. Within the realm of possible explanations, MIS-C might have arisen as a consequence of either Covid-19 vaccination (MIS-V) (2–6) or infection (7–13). Additionally, Kikuchi-Fujimoto Disease (KFD) has been linked to Covid-19 vaccination (14–22) and infection (23–29). Nevertheless, the pathological examination of the lymph node did not align with KFD. It remains conceivable that either infection-related or vaccine-related MIS could elucidate the colonic inflammation observed, characterized by ulceration, necrosis, and lymphadenitis of the terminal ileum.

### MIS-C or MIS-V: navigating diagnostic complexity in postoperative setting

The post-operative trajectory of the patient introduced a diagnostic conundrum characterized by persistent fever, diarrhea, rash, and conjunctivitis within the context of a recently resected colonic mass of uncertain implications. Given the patient's Covid-19 exposure 5 weeks prior to admission and Covid-19 vaccination administered at 10 and 6 weeks before admission, the management strategy encompassed considerations for both MIS-C and MIS-V.

MIS-C, an infrequent and novel hyperinflammatory disorder, surfaces 2–6 weeks subsequent to Covid-19 infection or exposure (30–32). Its diagnostic criteria involve elevated fever and multi-system inflammation, impacting at least two organ systems: cardiac involvement marked by hypotension, myocarditis, and coronary artery aneurysms; gastrointestinal manifestations comprising diarrhea, abdominal pain, and vomiting; mucocutaneous involvement indicated by skin rash, oral mucosal or conjunctival inflammation; shock; hematologic involvement such as thrombocytopenia or lymphopenia. Notably, the presentation of MIS-C often bears similarities to other hyperinflammatory syndromes including Kawasaki Disease (KD), toxic shock syndrome, and macrophage activation syndrome.

In a pre-MIS-C era, the patient's postoperative presentation would have raised concerns about KD, a medium vessel inflammatory vasculitis. KD diagnostic criteria involve a fever duration surpassing 4 days and distinct clinical characteristics, namely: (1) polymorphic skin rash, (2) involvement of lips and oral mucosa, (3) unilateral cervical lymphadenopathy, (4) non-exudative bilateral conjunctivitis, and (5) palmar/plantar erythema and swelling. Significantly, 25%–50% of MIS-C patients meet the complete criteria for KD diagnosis (30). The differentiation between the two conditions can be challenging without Covid-19 exposure or infection as a distinguishing factor. The patient, for various reasons, fulfilled the MIS-C criteria: its age range (1.6–20 years) with a median age of 6–11 years, which contrasts KD that primarily affects children under 5 years; gastrointestinal involvement, more prevalent in MIS-C and occasionally resembling appendicitis, occasionally necessitating exploratory laparotomy, but less so in KD (30). Furthermore, the patient's MIS-C-specific laboratory findings included thrombocytopenia, neutrophilia, lymphopenia, hyponatremia, and coagulopathy abnormalities with elevated D-dimer and fibrinogen levels. Noteworthy differences encompass higher cardiac involvement in MIS-C, presenting as coronary artery dilation (14%–48% incidence rate), and a higher occurrence of shock (40%–80%) compared to KD (10%) (30).

The pathogenesis of MIS-C likely involves a blend of post-infectious immune dysregulation, virus-induced cytopathic effects, and inflammation across multiple organ systems. The SARS-CoV-2 viral spike protein might serve as a superantigen, inciting a cytokine storm that precipitates MIS-C (33). Patients with Covid-19 and MIS-C exhibit higher IL-17 expression compared to those with Covid-19 alone; similarly, elevated levels of IL-10 and TNF-α are seen in MIS-C compared to severe Covid-19 infection (33). Another study highlights increased IL-1β, IL-6, IL-8, IL-10, IL-17, and IFNγ levels, coupled with diminished T and B cell subsets in the acute phase of MIS-C patients (31). The adaptive immune system's role is pivotal in MIS-C, driven by anti-SAR-COV-2 antibodies or SAR-COV-2 induced *de novo* autoantibodies (33). The treatment landscape for MIS-C involves supportive measures, IVIG, and steroids. Refractory cases might necessitate IL-1 receptor antagonist (Anakinra), TNF-α blockade (Infliximab), and IL-6 receptor antagonist (Tocilizumab) (31).

### Kikuchi-Fujimoto disease (KFD)

KFD is a rare, idiopathic, and generally benign cause of histiocytic necrotizing lymphadenitis. KFD typically presents with acute or subacute lymph node swelling, fatigue, headache, erythematous rashes, and hepatosplenomegaly; laboratory findings often include leukopenia, anemia, elevated inflammation markers and lactate dehydrogenase which have historically led to misdiagnosis in around 30% of patients initially suspected of having malignant lymphoma (34). There's also a connection between KFD and autoimmune disorders, with systemic lupus erythematosus being the most common.

The diagnosis of KFD is established through clinical assessment and histopathology. Lymph node biopsy typically reveals distorted architecture with cortical and paracortical nodules, histiocytic necrosis, and an absence of granulocytes, which were not observed in the presented case. Viral lymphadenitis can sometimes mimic KFD, sharing histologic features like paracortical expansion, necrosis, and histiocytic infiltrate. However, viral lymphadenitis typically displays less prominent histiocytic infiltrate, more neutrophils, plasma cells, and a predominant CD4 T cell population (35).

### Lymph node involvement in KFD and MIS-C

In KFD, lymph node involvement typically manifests in the cervical region, however, abdominal, pelvic, inguinal, axillary, and mediastinal lymphadenopathy can also occur (36). In contrast, lymph node involvement in MIS-C is less common and is primarily observed as cervical lymphadenopathy (33). In both KFD and MIS-C, mesenteric lymphadenopathy is rare, with only a few reported cases documented in the literature (35, 37).

### Lymph node involvement in adenovirus infection

In a comprehensive study encompassing more than 400 cases of acute infantile gastroenteritis, enteric adenoviruses emerged as the exclusive identifiable cause of diarrhea in 7.2 percent of instances (38). Beyond its association with gastroenteritis, adenovirus has been implicated in mesenteric adenitis and ileocecal intussusception. Notably, adenovirus can manifest clinically, mimicking the symptoms of acute appendicitis. A retrospective analysis further revealed that, among 94 patients clinically diagnosed with acute appendicitis, adenovirus infection was discerned upon pathological examination of the appendix in two pediatric cases. This underscores the diverse clinical manifestations of adenovirus and its potential to mimic other gastrointestinal conditions. Nevertheless, there is a notable absence of case reports in the English literature documenting giant abdominal lymph node in association with adenovirus gastroenteritis and pathologic feature of lymph node is different than post-infectious inflammatory disorders (35).

### MIS-C mimicking appendicitis

The patient in this case initially presented with abdominal pain suggestive of acute appendicitis but was later diagnosed with necrotizing mesenteric lymphadenitis and subsequently treated for MIS-C. It's noteworthy that MIS-C can sometimes present with abdominal symptoms that closely resemble appendicitis.

A retrospective analysis conducted by Vanseviciene et al. categorized children with acute abdominal pain into four groups: those with acute appendicitis, MIS-C with acute appendicitis, MIS-C alone, and acute appendicitis with Covid-19 (39). The study discovered that MIS-C can be accurately predicted with a high sensitivity (94%) and specificity (92.7%) when three of the following four criteria are met: elevated CRP levels exceeding 55.8 mg/L, gastrointestinal symptoms persisting for at least 3 days, presence of fever, and involvement of other organ systems, especially in individuals with recent Covid-19 infection. Approximately 8% of the patients in the study had MIS-C in combination with acute appendicitis.

Patients with MIS-C and appendicitis often exhibit coagulopathy, experience right lower quadrant pain, and display lymph node hyperplasia. Neutrophilia is commonly observed in cases of MIS-C with appendicitis, while a normal white blood cell count is seen in cases without appendicitis. Ultrasound visualization of the appendix is notably more pronounced in MIS-C with appendicitis, featuring a thicker appendiceal diameter, whereas the opposite is true for MIS-C alone, with lower appendix visibility and a smaller diameter. Appendicitis resulting from viral infection is likely attributed to hypertrophy of lymphoid tissue in the appendiceal wall, leading to lumen obstruction or direct inflammation within the lymphoid tissue. Covid-19 infection may enter the appendix through the ACE2 receptor on enterocytes, similar to the ileum. Patients with MIS-C and complicated appendicitis often exhibit elevated levels of proinflammatory cytokines (39).

It's crucial to recognize MIS-C early in patients whose symptoms resemble those of a surgical abdomen to avoid unnecessary surgeries. Some patients with concomitant MIS-C and appendicitis may require treatment for MIS-C prior to undergoing surgery. Laboratory criteria, such as a low-normal white blood cell count and thrombocytopenia, can support a diagnosis of MIS-C in patients presenting with appendicitis-like symptoms and a positive Covid-19 IgG (37).

Radiologically, MIS-C can mimic acute appendicitis on CT imaging. Abdominal imaging in MIS-C may reveal hepatomegaly, nephromegaly, gallbladder wall thickening, ascites, mesenteric lymphadenopathy, and increased renal echogenicity (40).

**Figure 4:**
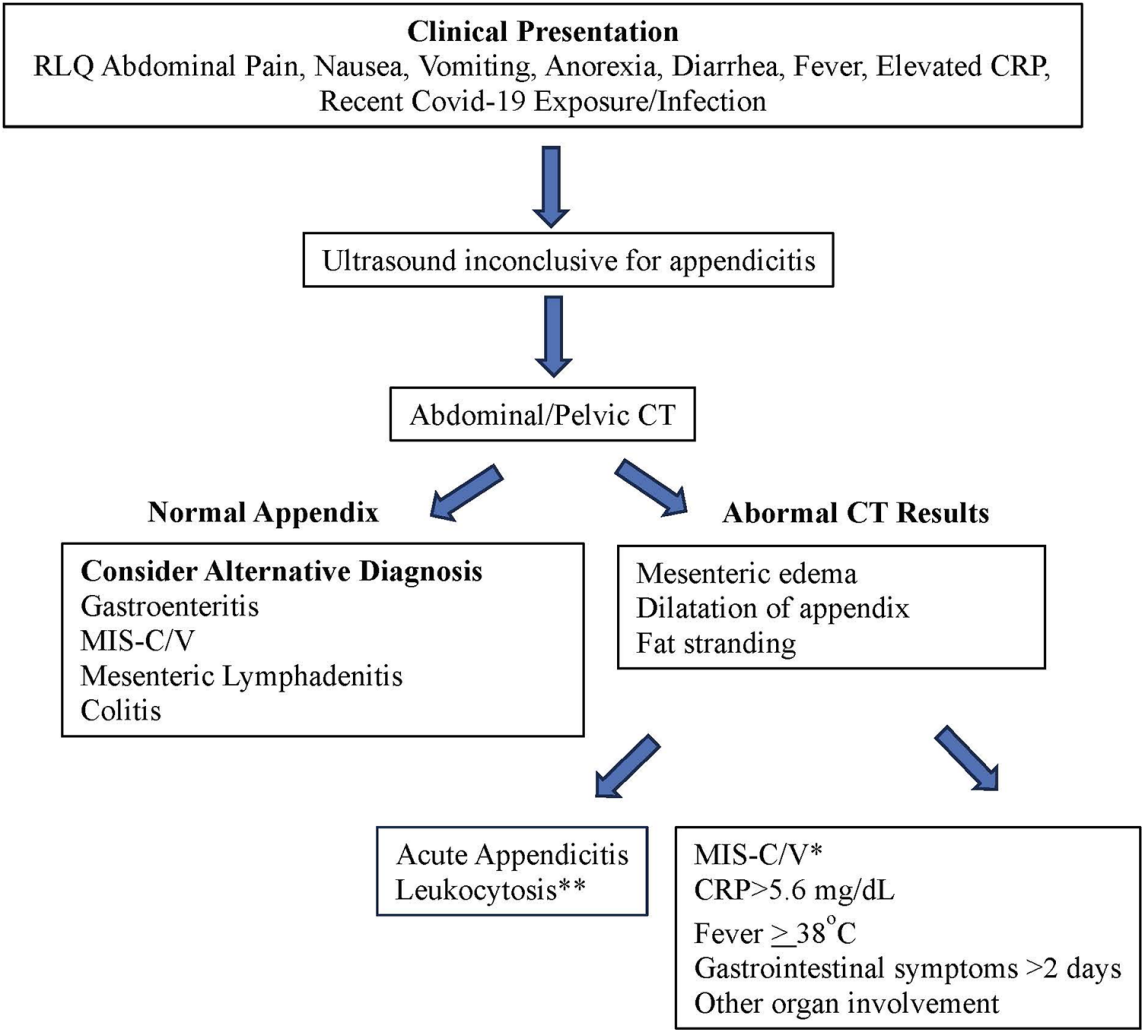
Differentiating acute bacterial appendicitis from MIS-C. *Predictive of MIS-C with sensitivity of 94.3% and specificity of 92.7% (38). **Appendicitis is more likely if WBC > 14 × 10^9^/L.

In patients with acute abdomen suspicious for appendicitis, the differential diagnosis should encompass infectious and postinfectious inflammatory and malignant disorders such as bacterial appendicitis, MIS-C/V, KFD, and mesenteric malignant lymphadenopathy. Scoring systems based on clinical, laboratory, and imaging criteria have been developed to predict or rule out acute appendicitis in children (41–43), but it's essential for clinicians to also consider MIS-C and KFD as potential masqueraders of appendicitis, especially during the ongoing Covid-19 pandemic, which has had a lasting impact on healthcare practices. We propose a differential diagnostic flow chart to aid in the differentiation of acute bacterial appendicitis from MIS-C (Figure 4), and clinical, laboratory and radiologic features of various differential diagnoses of MIS-C relative to other conditions with overlapping symptoms (Table 1) (34, 40, 44, 45).

**Table 1:** Clinical, laboratory and radiologic features of various differential diagnoses of MIS-C relative to other conditions with overlapping symptoms (34, 40, 44, 45).

	Acute appendicitis	Acute appendicitis with MIS-C	MIS-C	Acute appendicitis with Covid-19	COVID(−) Kawasaki	COVID(+) Kawasaki or MIS-C	Kikuchi-Fujimoto
Age	Any	Older	Older	Any	Younger	Older	Older
Abdominal pain	+++	++	++	++	+	+++	−
Tender RLQ	+++	++	−	+	−	−	−
Fever	++	+++	+++	+/−	+++	+++	++
Abdominal muscle rigidity	++	+/−	−	+	−	−	−
≥2 Organ involvement	−	+++	+++	−	++	+++	−
Cervical LAD	−	−	+/−	−	++	+/−	+++
Appendicitis in abdominal ultrasound/CT	+++	+	−	++	−	−	−
Shock	−	+	++	−	+/−	+	−
Mesenteric LAD in AUS	−	+	+	−	−	+	+
CRP	+	+++	+++	−	+++	+++	+++
Anemia	−	+	+	−	++	+	−
High WBC	+	−	−	−	++	−	−
Lymphopenia	−	+++	+++	−	−	+++	++
Low platelets	−	++	++	−	−	++	+
Low albumin	−	++	++	−	+	++	+/−
High ferritin	+/−	++	++	−	+	++	+/−
High D-dimer	−	++	++	−	+	++	−
High BNP/Troponin	−	++	++	−	+	++	−
Coronary artery involvement	−	+/−	+/−	−	++	+/−	−

(−), Unusual; (+/−), rare; (+), 10%–30%; (++), 40%–60%; (+++), 70%–100%. RLQ, right lower quadrant; LAD, lymphadenopathy; CT, computerized tomography; BNP, brain natriuretic protein.

The absence of conclusive evidence regarding the antibody response to COVID-19 vaccines may be considered a limitation in our case report. However, the lack of a history of recurrent or unusual infections, which might suggest immune deficiency, and the absence of immunosuppressive therapy significantly diminish the likelihood of antibody unresponsiveness. The rapid and positive response to IVIG, along with the complete healing observed at the 1-month follow-up, provides strong support for the accuracy of our diagnosis and the efficacy of the chosen treatment. Furthermore, the patient's sustained asymptomatic status over the course of a year adds a compelling layer of evidence, further reinforcing the strength and reliability of our clinical decisions.

## Conclusion

Clinicians should keep MIS-C/V and KFD in mind when diagnosing acute abdominal cases. The presented case underscores the importance of recognizing atypical presentations of MIS-C that can mimic appendicitis, sometimes necessitating surgical resection of a large lymph node. We propose diagnostic flow chart to aid in the differentiation of acute bacterial appendicitis from MIS-C.

## Data Availability

The data analyzed in this study is subject to the following restrictions: Case report includes patient's clinical and laboratory data. Requests to access these datasets should be directed to the corresponding author.
